# Microbial community profiles of the colon from steers differing in feed efficiency

**DOI:** 10.1186/s40064-015-1201-6

**Published:** 2015-08-27

**Authors:** Phillip R Myer, James E Wells, Timothy P L Smith, Larry A Kuehn, Harvey C Freetly

**Affiliations:** USDA, ARS, U.S. Meat Animal Research Center, Clay Center, NE 68933 USA; Department of Animal Science, The University of Tennessee, Knoxville, TN USA

**Keywords:** 16S rRNA, Colon, Feed efficiency, Microbiome, Operational taxonomic units

## Abstract

Ruminal microbial fermentation plays an essential role in host nutrition, and as a result, the rumen microbiota have been a major focus of research examining bovine feed efficiency. Microbial communities within other sections of the gastrointestinal tract may also be important with regard to feed efficiency, since it is critical to the health and nutrition of the host. The objective of this study was to characterize the microbial communities of the colon among steers differing in feed efficiency. Individual feed intake (FI) and body weight (BW) gain were determined from animals fed the same ration, within two contemporary groups of steers. Four steers from each contemporary group within each Cartesian quadrant were sampled (*n* = 16/group) from the bivariate distribution of average daily BW gain and average daily FI. Bacterial 16S rRNA gene amplicons were sequenced from the colon content using next-generation sequencing technology. Within the colon content, UniFrac principal coordinate analyses did not detect any separation of microbial communities, and bacterial diversity or richness did not differ between efficiency groups. Relative abundances of microbial populations and operational taxonomic units did reveal significant differences between efficiency groups. The phylum Firmicutes accounted for up to 70% of the populations within all samples, and families Ruminococcaceae and Clostridiaceae were highly abundant. Significant population shifts in taxa were detected, including the families Ruminococcaceae, Lachnospiraceae, and Sphingomonadaceae, and the genera *Butyrivibrio*, *Pseudobutyrivibrio*, *Prevotella*, *Faecalibacterium* and *Oscillospira*. This study suggests the association of the colon microbial communities as a factor influencing feed efficiency at the 16S level.

## Background

Feed costs remain the largest variable cost in beef production (Arthur et al. 2005). Although it has been estimated that a 10% improvement in performance (gain) would increase profitability by only 18%, increasing the efficiency of growth of feedlot cattle by 10% could improve profitability by upwards of 43% (Fox et al. 2001). Optimization of feed efficiency in beef cattle is also important for social and environmental issues, due to increasing concerns over methane emissions of cattle, decreasing acreage for crop production (Wirsenius et al. 2010) increasing world population, and increasing divergence of traditional livestock feedstuffs for production of biofuels (Galyean et al. 2011). Genetic selection promises a lasting approach to optimizing the food produced and feed consumed per animal, but traditional selection requires costly phenotyping of individuals. A combination of traditional nutritional and management approaches, in tandem with genetic improvement of feed efficiency in beef cattle, represents a potential path to sustainably reduce feed resources required to produce beef, as well as contribute towards the environmental sustainability of beef production.

The study and improvement of feed efficiency in beef cattle has primarily focused on host-related genetic improvement technologies; however, host genetic contributions to feed efficiency in beef cattle have been difficult to identify (Saatchi et al. 2014; Sherman et al. 2010; Abo-Ismail et al. 2014). The gastrointestinal tract (GIT) contains complex and dynamic microbial communities, which have long been regarded as essential in structure, function, and overall health of the host (Fujimura et al. 2010). Among other contributing factors to beef cattle nutrition, such as host genetics, diet, and management, the microbial populations within the GIT must also be examined to comprehensively evaluate their effect on feed efficiency. Furthermore, microbial-associated feed efficiency studies have largely concentrated on ruminal associations, due to the role of the rumen in nutrient supply to the host (Kim et al. 2011; Hernandez-Sanabria et al. 2012; Jami et al. 2014; McCann et al. 2014). Myer et al. (2015) demonstrated associations between specific microbial populations and feed efficiency phenotypes in beef cattle. However, bovine ruminal microbial communities are distinct from those of the colon and feces (de Oliveira et al. 2013), therefore a full understanding of the relationship between the microbial populations along the GIT and feed efficiency, gain, and intake, requires characterization of sites distal from the rumen.

High-throughput sequencing technologies have aided researchers in the examination of microbial communities, enabling the study of the structure and function of microbial populations at great depth, and revealing significant differences within microbial communities that would not otherwise be detectable using culture-based methodologies. High-throughput molecular technologies are able to resolve complex microbial communities at finer resolutions, providing the opportunity to identify the relationships between microbial community structure and feed efficiency phenotypes.

The objective of this study was to examine the association between microbial community structure and feed efficiency phenotypes within the lower GIT, by determining the microbial community of the colon from steers differing in feed efficiency using deep 16S rRNA-based community profiling. We hypothesize that variation in the microbial populations within the colon impact or reflect variation in host feed efficiency. This study aimed to characterize the bacterial community of the cattle colon among steers differing in feed intake and growth, in order to assess the association of the microbial community profile with variation in bovine feed efficiency.

## Results

### Diversity of colon bacterial communities

The colon contents sampled from the 32 steers grouped into 4 feed efficiency phenotypes, produced 20,593,775 sequence reads after filtering for quality and removing apparent chimeras, for an average of 682,061 reads per sample (range 110,537–2,668,201). The average read length was 500 bp. OTUs were defined as a bin of sequence reads sharing ≥97% nucleotide sequence identity. From the cleaned sequences, a total of 323,433 OTUs were detected with an average of 10,107 ± 2,896 OTUs per individual sample. The average number of OTUs detected from each Cartesian quadrant ranged from 7,247 to 15,130 OTUs. Singletons accounted for approximately 38% of the OTUs detected within the colon content samples. The dataset reported coverage ranging from 95.82 to 99.16%, using Good’s coverage estimator as a metric for determining coverage. Bacterial diversity, as determined by Shannon diversity index, ranged from 6.84 to 8.93.

The individual samples were normalized in order to accurately compare among feed efficiency phenotype groups. The OTU table within each sample was rarefied to 100,000 sequence reads, based upon the sample rarefaction curves. The normalized samples were then used for analysis using the sample means within each quadrant. The normalized sequence reads were analyzed via alpha-diversity metrics of bacterial diversity (Shannon Index), richness (Chao-1), evenness (equitability of representation of taxa), and coverage (Good’s coverage estimator; Table 1). The number of OTUs detected within each feed efficiency group did not differ (*P* > 0.05), averaging 6,025 ± 1,225 OTUs per group. The Chao-1 richness metric also did not differ, estimating 10,051 ± 2,334 OTUs per group. Bacterial diversity did not indicate any differences between feed efficiency groups (*P* > 0.05), with a range of 7.85–8.27. Evenness, as estimated by equitability (1 = complete equitability), did not differ between groups (*P* > 0.05), averaging 0.59 ± 0.03 per group. Coverage was adequate, ranging from 96.53 to 97.13%, representative of the ADG_low_–ADFI_low_ and ADG_high_–ADFI_high_ groups, respectively.

**Table 1:** Diversity statistics among reads from grouped samples

Feed efficiency group	Sampling type	No. of sequences	No. of OTUs^a,b^	Chao1^b^	Shannon diversity index^b^	Equitability^b^	Good’s coverage (%)
ADG_high_–ADFI_high_^c^	Subsampled reads^d^	100,000	5,764 ± 878	9,638 ± 2,004	7.85 ± 0.52	0.58 ± 0.02	97.13 ± 0.59
ADG_high_–ADFI_low_^c^	Subsampled reads^d^	100,000	6,098 ± 1,028	9,874 ± 1,853	8.15 ± 0.39	0.60 ± 0.02	97.02 ± 0.60
ADG_low_–ADFI_low_^c^	Subsampled reads^d^	100,000	6,714 ± 1,148	11,538 ± 2,333	8.27 ± 0.49	0.60 ± 0.03	96.53 ± 0.71
ADG_low_–ADFI_high_^c^	Subsampled reads^d^	100,000	6,063 ± 1,129	10,052 ± 2,003	7.92 ± 0.74	0.59 ± 0.02	96.99 ± 0.61

^a^OTUs represents operational taxonomic units.

^b^Within a column, means for the individual subsamples did not differ (*P* < 0.05).

^c^ *n* = 8 among groups.

^d^Means among the groups were compared using ANOVA and the Tukey’s test.

**Fig. 1:**
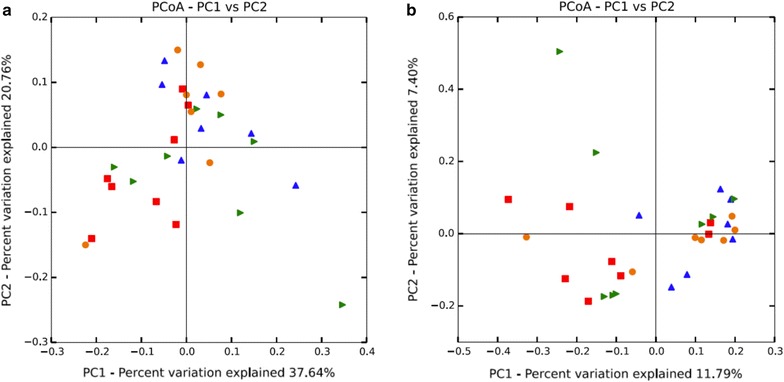
UniFrac principal coordinates analysis (PCoA) displaying correlations among the bacterial communities of the 4 feed efficiency groups. **a** Weighted PCoA analyzed from rarefied subsets of 100,000 sequences from each sample. **b** Unweighted PCoA analyzed from rarefied subsets of 100,000 sequences from each sample. *n* = 8, represented by differing *symbols*: ADG_High_–ADFI_High_ *orange circle*, ADG_High_–ADFI_Low_ *blue triangle*, ADG_Low_–ADFI_Low_ *red square*, ADG_Low_–ADFI_High_ *green triangle*.

Principal coordinates analyses (PCoA) were conducted to determine the phylogenetic relationship between microbial community samples in the study (Fig. 1). The PCoA utilizes the phylogeny-based UniFrac method, which uses the detected OTUs to determine if the data separate into any sample clusters. This beta-diversity metric takes into account the phylogenetic divergence between the OTUs, in order to determine differences within the colonic microbial communities from each feed efficiency group (Lozupone et al. 2007). The analysis did not indicate any separation into clusters in both the weighted (quantitative) and unweighted (qualitative) UniFrac distances of the colon microbial communities (Lozupone et al. 2011).

### Taxonomic and OTU composition

**Fig. 2:**
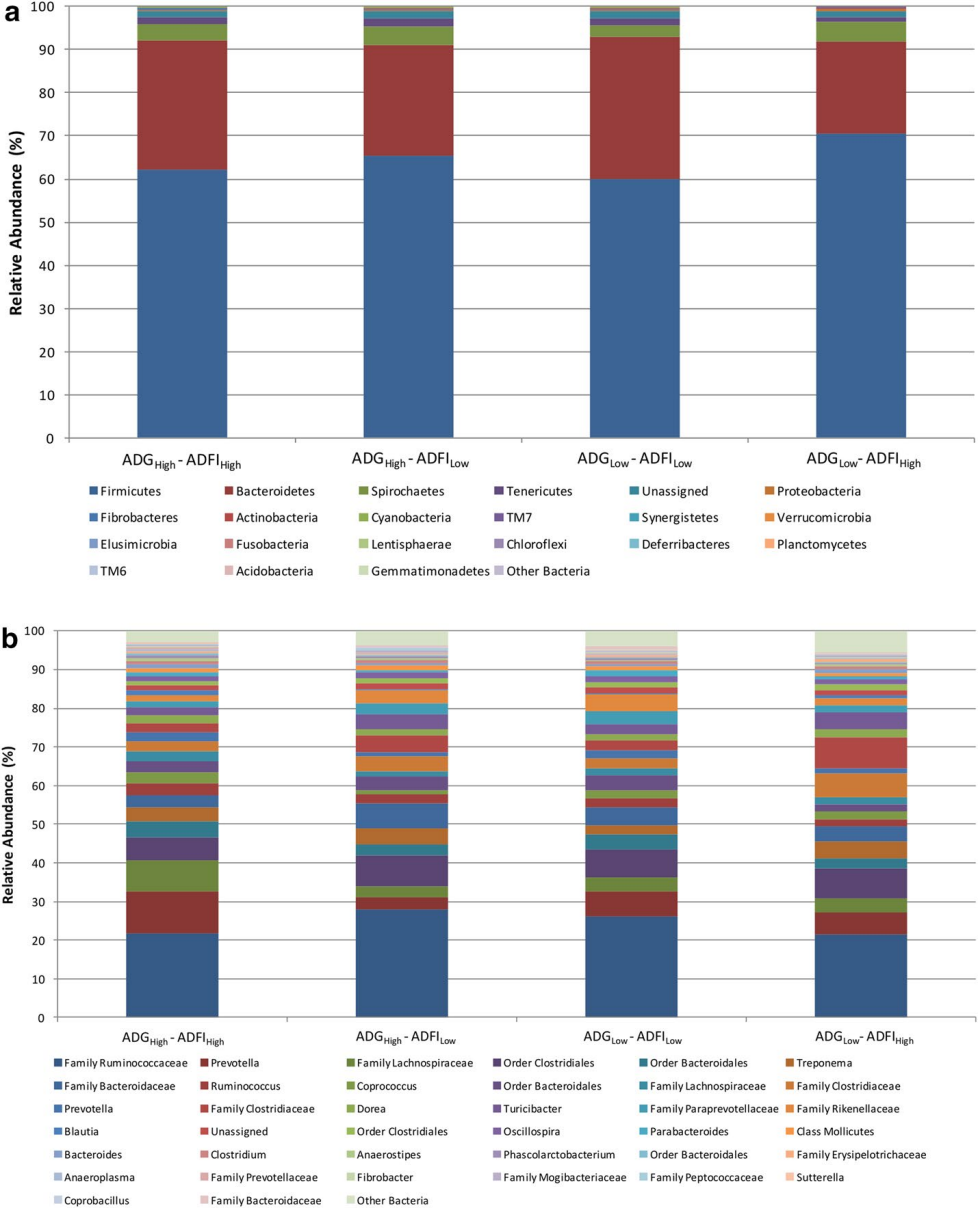
The taxonomic profiles for the relative phylum-level (**a**) and genus-level (**b**) abundance of each group classified by representation at ≥0.001% of total sequences. Taxonomic composition of the colon microbiota among the four groups was compared based on the relative abundance (reads of a taxon/total reads in a sample).

The 20,593,775 amplicon sequence reads were classified using the Greengenes 16S rRNA Gene Database (DeSantis et al. 2006) resulting in 20 phyla, 46 classes, 83 orders, 152 families, and 231 genera. The unassigned taxa accounted for approximately 1.48% of the reads. Firmicutes was the most abundant phylum within each feed efficiency group, ranging from 60 to 70% (in terms of percent of the total reads; Fig. 2a). These abundances are consistent with previous studies regarding the microbial abundances within the colon/fecal contents of cattle (de Oliveira et al. 2013; Malmuthuge and Griebel 2014). Other dominant phyla included Bacteroidetes (21–33 ± 3.4%), Spirochaetes (2.5–4.5 ± 1.3%), Tenericutes (1.2–1.9 ± 0.4%), Proteobacteria (0.3–0.5 ± 0.9%), Actinobacteria (0.23–0.33 ± 0.1%), and Fibrobacteres (0.02–0.29 ± 0.2%). No significant differences between the feed efficiency groups were observed within any of the phylum assignments. The remaining phyla accounted for <0.1% of the sequence reads, and no differences were observed between feed efficiency groups for the minor phyla abundances.

At the genus level, *Prevotella* (3.0–11.1 ± 2.7%), *Ruminococcus* (1.7–2.9 ± 0.4%), *Coprococcus* (1.0–2.9 ± 0.5%), *Dorea* (1.7–2.2 ± 0.3%), *Turicibacter* (1.9–4.4 ± 0.8%), *Blautia* (0.3–1.3 ± 0.2%), *Oscillospira* (1.1–1.6 ± 0.2%), and *Parabacteroides* (0.4–1.4 ± 0.3%) were present at greatest abundance, representing ≥1% of the total sequences (Fig. 2b). Of these genera, only *Prevotella* differed among the feed efficiency groups (*P* = 0.0259), with the ADG_High_–ADFI_High_ group having the greatest abundance (Table 2). There were several taxa that were not classified to the genus level, but were present in abundances greater than 1% of the total sequences. These taxa included families Ruminococcaceae (21–28 ± 2.2%), Lachnospiraceae (2.9–7.9 ± 1.2%), Bacteroidaceae (3.3–6.4 ± 1.4%), Clostridiaceae (2.6–6.1 ± 1.1%), Paraprevotellaceae (1.6–3.4 ± 0.9%), and Rikenellaceae (1.5–4.2 ± 0.8%), as well as orders Clostridiales (6.0–7.9 ± 1.1%) and Bacteroidales (2.5–3.9 ± 0.6%). None of the aforementioned taxa at this level differed among the feed efficiency groups. Any remaining taxa were not listed and deemed non-detectable at abundances ≤ 0.001%.

**Table 2:** Relative abundance of significant taxa in the four feed efficiency groups

Classification	Percentage of sequences^a^				SEM	*P* value^b^	No. of steers with detectable taxon^c^
	ADG_High_–ADFI_High_	ADG_High_–ADFI_Low_	ADG_Low_–ADFI_Low_	ADG_Low_–ADFI_High_			
*Anaeroplasma*	0.1587	0.0591	0.0652	0.0854	0.0230	0.0222	30
Cyanobacteria	0.0187	0.0103	0.0113	0.0093	0.0024	0.0479	29
*Faecalibacterium*	0.1976	0.0276	0.0357	0.0916	0.0419	0.0361	23
Family Barnesiellaceae	2.49 × 10^−4^	0.0192	0.0216	9.93 × 10^−4^	0.0063	0.0471	24
Family Mogibacteriaceae	0.1013	0.1611	0.0762	0.2295	0.0358	0.0309	30
Family Sphingomonadaceae	0.0012	0.0011	2.38 × 10^−4^	1.24 × 10^−4^	3.11 × 10^−4^	0.0386	29
*Paludibacter*	1.23 × 10^−4^	4.86 × 10^−5^	0.0028	9.94 × 10^−4^	6.29 × 10^−4^	0.0226	27
*Prevotella*	7.9657	3.1272	3.9112	3.5961	1.1520	0.0259	30
*Pseudobutyrivibrio*	0.0187	0.0103	0.0113	0.0093	0.0024	0.0479	21
*Succinivibrio*	0.0090	9.76 × 10^−4^	0.0020	0.0037	0.0020	0.0412	29

^a^Data is shown as LSMeans.

^b^ *P* values indicate groups that differ (P < 0.05).

^c^All data are defined as taxa that are present in at least 50% of the samples.

Additional taxa were identified at low relative abundances, and differences were detected between feed efficiency phenotypes. These taxa included the genera *Anaeroplasma* (*P* = 0.0222), *Paludibacter* (*P* = 0.0226), *Faecalibacterium* (*P* = 0.0361), *Succinivibrio* (*P* = 0.0412), and *Pseudobutyrivibrio* (*P* = 0.0479). Of these taxa, *Anaeroplasma* and *Faecalibacterium* were in greatest abundance within the ADG_High_–ADFI_High_ group, *Paludibacter* was in greatest abundance within the ADG_Low_–ADFI_Low_ group, and *Succinivibrio* and *Pseudobutyrivibrio* were least abundant within the ADG_High_–ADFI_Low_ and ADG_Low_–ADFI_High_ groups, respectively (Table 2). Differences between the groups were also detected within other low abundance taxa, but not classified to the genus level. These low-abundance taxa included identifications within the families Mogibacteriaceae (*P* = 0.0309), Sphingomonadaceae (*P* = 0.0386), and Barnesiellaceae (*P* = 0.0471), with the greatest abundance within the ADG_Low_–ADFI_High_ group for the family Mogibacteriaceae. The families Sphingomonadaceae and Barnesiellaceae were least abundant within the ADG_Low_–ADFI_Low_ and ADG_Low_–ADFI_High_ groups, respectively (Table 2). The taxa listed were observed as present in at least 50% of the samples.

The examination of OTUs across all feed efficiency phenotype groups was also conducted to detect differences in abundance. Consideration was only given to OTUs detectable at abundances > 0.001% and present in at least 50% of the samples. Among the groups, 68 OTUs were identified that differed in abundance (Table 3). The most common and functionally significant OTUs identified with families Ruminococcaceae (*P* = 0.0029), Lachnospiraceae (*P* = 0.0279), and Clostridiaceae (*P* = 0.0460), as well as the orders Clostridiales (*P* = 0.0264) and Bacteroidales (*P* = 0.0433). At the genus level, *Dorea* (*P* = 0.0225), *Butyrivibrio* (*P* = 0.0240), *Coprococcus* (*P* = 0.0323), *Prevotella* (*P* = 0.0435), *Clostridium* (*P* = 0.0446), *Oscillospira* (*P* = 0.0456), and *Pseudobutyrivibrio* (*P* = 0.0476), as well as the species *Prevotella copri* (*P* = 0.0483) differed between groups. *Coprococcus*, *Clostridium*, *Pseudobutyrivibrio*, and *Prevotella copri* were all in greatest abundance within the ADG_High_–ADFI_High_ group, while *Dorea* was least abundant within this group. The abundances of *Butyrivibrio* and *Prevotella* were greatest within the ADG_Low_–ADFI_High_ group. Finally, the abundance of *Oscillospira* was greatest within the ADG_Low_–ADFI_Low_ group (Table 3).

**Table 3:** Relative abundance of significant OTUs in the four feed efficiency groups

OTU ID	Classification	Percentage of total sequences^a^				SEM	*P* value^b^	No. of steers with detectable taxon^c^
		ADG_High_–ADFI_High_	ADG_High_–ADFI_Low_	ADG_Low_–ADFI_Low_	ADG_Low_–ADFI_High_			
denovo26989	*Bacteroides*	0.0163	0.0026	0.0013	0.0040	0.0034	0.0475	17
denovo77346	*Blautia*	0.0028	0.0003	0.0007	0.0014	0.0006	0.0499	19
denovo8562	*Butyrivibrio*	0.0003	0.0021	0.0009	0.0029	0.0004	0.0240	20
denovo24149	*Butyrivibrio*	0.0003	0.0016	0.0005	0.0018	0.0004	0.0471	16
denovo8243	*Clostridium*	0.0298	0.0018	0.0022	0.0009	0.0066	0.0446	23
denovo8936	*Coprococcus*	0.0063	0.0017	0.0011	0.0010	0.0009	0.0323	15
denovo67746	*Coprococcus*	0.0061	0.0014	0.0029	0.0009	0.0011	0.0441	19
denovo33574	*Dorea*	0.0001	0.0010	0.0010	0.0025	0.0003	0.0225	18
denovo12537	Family Bacteroidaceae	0.0001	0.0019	0.0009	0.0013	0.0004	0.0500	15
denovo66919	Family Christensenellaceae	0.0043	0.0086	0.0030	0.0015	0.0014	0.0442	23
denovo66901	Family Christensenellaceae	0.0021	0.0121	0.0109	0.0029	0.0024	0.0463	18
denovo28680	Family Clostridiaceae	0.0086	0.0010	0.0016	0.0019	0.0017	0.0460	19
denovo23600	Family Lachnospiraceae	0.0641	0.0126	0.0175	0.0210	0.0086	0.0279	29
denovo25904	Family Lachnospiraceae	0.0018	0.0023	0.0039	0.0009	0.0006	0.0472	23
denovo67326	Family Lachnospiraceae	0.0024	0.0005	0.0012	0.0005	0.0004	0.0482	16
denovo43262	Family Lachnospiraceae	0.0084	0.0008	0.0025	0.0010	0.0018	0.0487	17
denovo6452	Family Lachnospiraceae	1.5328	0.1368	0.1559	0.0641	0.3227	0.0495	30
denovo37795	Family Lachnospiraceae	0.0021	0.0016	0.0007	0.0039	0.0007	0.0496	21
denovo25335	Family Lachnospiraceae	0.0085	0.0028	0.0022	0.0028	0.0017	0.0499	23
denovo13622	Family Peptostreptococcaceae	0.0001	0.0019	0.0013	0.0011	0.0004	0.0499	16
denovo34577	Family Rikenellaceae	0.0009	0.0067	0.0384	0.0033	0.0077	0.0485	18
denovo11639	Family Rikenellaceae	0.0155	0.0367	0.0567	0.0185	0.0104	0.0497	26
denovo50904	Family Ruminococcaceae	0.0013	0.0063	0.0033	0.0005	0.0007	0.0029	20
denovo53814	Family Ruminococcaceae	0.0045	0.0011	0.0013	0.0008	0.0006	0.0246	19
denovo11996	Family Ruminococcaceae	0.0004	0.0004	0.0019	0.0004	0.0003	0.0387	15
denovo43427	Family Ruminococcaceae	0.0044	0.0010	0.0022	0.0009	0.0007	0.0416	20
denovo6911	Family Ruminococcaceae	0.0061	0.0011	0.0014	0.0018	0.0010	0.0421	22
denovo71254	Family Ruminococcaceae	0.1496	0.5316	0.4293	0.2538	0.0697	0.0440	30
denovo9573	Family Ruminococcaceae	0.0023	0.0076	0.0133	0.0044	0.0019	0.0445	21
denovo19097	Family Ruminococcaceae	0.0024	0.0160	0.0135	0.0075	0.0028	0.0447	26
denovo30835	Family Ruminococcaceae	0.0005	0.0010	0.0021	0.0003	0.0004	0.0448	15
denovo46467	Family Ruminococcaceae	0.0023	0.0320	0.0111	0.0033	0.0062	0.0458	20
denovo38547	Family Ruminococcaceae	0.0103	0.0078	0.0051	0.0019	0.0017	0.0459	27
denovo359	Family Ruminococcaceae	0.0345	0.0047	0.0100	0.0124	0.0065	0.0466	27
denovo19227	Family Ruminococcaceae	0.0006	0.0034	0.0029	0.0016	0.0006	0.0470	22
denovo38238	Family Ruminococcaceae	0.3534	0.8847	0.6177	0.6599	0.1075	0.0476	30
denovo25283	Family Ruminococcaceae	0.0228	0.0595	0.0320	0.0288	0.0078	0.0478	29
denovo67138	Family Ruminococcaceae	0.0105	0.0015	0.0023	0.0030	0.0020	0.0478	18
denovo39456	Family Ruminococcaceae	0.0011	0.0056	0.0016	0.0016	0.0008	0.0479	21
denovo41445	Family Ruminococcaceae	0.0025	0.0125	0.0049	0.0009	0.0023	0.0479	23
denovo16744	Family Ruminococcaceae	0.0019	0.0072	0.0031	0.0016	0.0012	0.0479	20
denovo69598	Family Ruminococcaceae	0.0008	0.0050	0.0016	0.0013	0.0009	0.0486	16
denovo21045	Family Ruminococcaceae	0.0043	0.0035	0.0012	0.0009	0.0008	0.0487	19
denovo21464	Family Ruminococcaceae	0.0020	0.0013	0.0068	0.0016	0.0012	0.0492	17
denovo59669	Family Ruminococcaceae	0.2908	0.2518	0.1567	0.0961	0.0449	0.0492	30
denovo44398	Family Ruminococcaceae	0.0034	0.0094	0.0184	0.0051	0.0033	0.0492	20
denovo70020	Family Ruminococcaceae	0.0008	0.0115	0.0065	0.0038	0.0025	0.0499	18
denovo37453	Order Bacteroidales	0.0040	0.0004	0.0008	0.0010	0.0006	0.0433	15
denovo46578	Order Bacteroidales	0.0018	0.1288	0.0686	0.0301	0.0264	0.0469	19
denovo19773	Order Bacteroidales	0.0126	0.0024	0.0028	0.0053	0.0022	0.0473	22
denovo72787	Order Bacteroidales	0.0184	0.0018	0.0042	0.0073	0.0035	0.0475	16
denovo8377	Order Bacteroidales	0.0043	0.0006	0.0013	0.0011	0.0008	0.0482	18
denovo29245	Order Bacteroidales	0.0069	0.0005	0.0011	0.0029	0.0016	0.0499	16
denovo40203	Order Clostridiales	0.0048	0.0006	0.0006	0.0008	0.0008	0.0264	16
denovo23255	Order Clostridiales	0.0011	0.0038	0.0015	0.0054	0.0008	0.0414	23
denovo75811	Order Clostridiales	0.0019	0.0021	0.0036	0.0068	0.0010	0.0454	27
denovo70498	Order Clostridiales	0.0018	0.0075	0.0060	0.0020	0.0013	0.0480	19
denovo13252	Order Clostridiales	0.0049	0.0103	0.0078	0.0149	0.0021	0.0485	29
denovo40361	Order Clostridiales	0.0054	0.0009	0.0021	0.0015	0.0010	0.0498	15
denovo60620	Order Clostridiales	0.0025	0.0077	0.0042	0.0015	0.0013	0.0499	21
denovo40059	Order Clostridiales	0.0008	0.0021	0.0018	0.0004	0.0005	0.0499	16
denovo18789	Order Clostridiales	0.0019	0.0066	0.0023	0.0071	0.0013	0.0499	25
denovo43715	Order Clostridiales	0.0185	0.0052	0.0080	0.0110	0.0032	0.0500	29
denovo51765	*Oscillospira*	0.0025	0.0046	0.0134	0.0041	0.0022	0.0456	25
denovo26903	*Prevotella*	0.0050	0.0017	0.0027	0.0179	0.0030	0.0435	28
denovo23534	*Prevotella copri*	0.0230	0.0011	0.0051	0.0033	0.0049	0.0483	17
denovo60154	*Pseudobutyrivibrio*	0.0064	0.0007	0.0017	0.0025	0.0012	0.0476	21
denovo45407	*Treponema*	0.0010	0.0237	0.0161	0.0049	0.0049	0.0477	21

^a^Data is shown as LSMeans.

^b^ *P* values indicate groups that differ (P < 0.05).

^c^Percentage of total sequences for steers with nondetectable OTUs were treated as 0.001%, and all data are defined as OTUs that are present in at least 50% of the samples.

### Effect of gain and intake

In order to examine the microbial population associations among the contributing factors of feed efficiency, the effect of the microbial communities on ADG and ADFI were analyzed separately to determine whether the associated microbial populations differed by low vs. high ADG, low vs. high ADFI, or their interaction. The significant relative abundances of taxa and OTUs between ADG and ADFI are listed in Tables 4 and 5, respectively. No taxa were associated with gain alone (Table 4), but two taxa were determined to have either a significant effect for intake or the interaction. When examined using OTUs, a majority of the OTUs associated with either intake or the interaction (Table 5). Pertaining to significant genera, *Bacteroides* (*P* = 0.0491) and *Blautia* (*P* = 0.0497) were associated with intake, while *Butyrivibrio* (*P* = 0.0156) and *Coprococcus* (*P* = 0.0499) were significant for the interaction. Additionally, the species *Prevotella copri* (*P* = 0.0491) associated with the interaction. OTUs classified within families Ruminococcaceae, Lachnospiraceae, and Clostridiaceae were associated with intake, as well as their interaction. The genera *Oscillospira* (*P* = 0.0484) and *Prevotella* (*P* = 0.0494) were the only classifications of OTUs associated solely with gain.

**Table 4:** Relative abundance of significant taxa within ADG and ADFI phenotypes

Classification	Phenotype^a^				Effect	SEM	*P* value^b^
	ADG_High_	ADG_Low_	ADFI_High_	ADFI_Low_			
*Prevotella*	5.5465	3.7537	5.7809	3.5192	Gain*Intake	0.8443	0.0484
*Succinivibrio*	0.0050	0.0028	0.0064	0.0015	Intake	0.0014	0.0411

^a^Data is shown as LSMeans.

^b^ *P* values indicate groups that differ (*P* < 0.05).

**Table 5:** Relative abundance of significant OTUs within ADG and ADFI phenotypes

OTU ID	Classification	Phenotype^a^				Effect	SEM	*P* value^b^
		ADG_High_	ADG_Low_	ADFI_High_	ADFI_Low_			
denovo26989	*Bacteroides*	0.0094	0.0027	0.0101	0.0020	Intake	0.0025	0.0491
denovo77346	*Blautia*	0.0015	0.0011	0.0021	0.0005	Intake	0.0004	0.0497
denovo8562	*Butyrivibrio*	0.0012	0.0019	0.0016	0.0015	Gain*Intake	0.0003	0.0156
denovo8936	*Coprococcus*	0.0040	0.0011	0.0036	0.0014	Gain*Intake	0.0007	0.0499
denovo28680	Family Clostridiaceae	0.0048	0.0017	0.0053	0.0013	Intake	0.0012	0.0486
denovo43262	Family Lachnospiraceae	0.0046	0.0017	0.0047	0.0016	Gain*Intake	0.0013	0.0494
denovo6452	Family Lachnospiraceae	0.8348	0.1100	0.7984	0.1464	Gain*Intake	0.2365	0.0496
denovo13622	Family Peptostreptococcaceae	0.0010	0.0012	0.0006	0.0016	Intake	0.0003	0.0492
denovo50904	Family Ruminococcaceae	0.0038	0.0019	0.0009	0.0048	Intake	0.0005	0.0034
denovo53814	Family Ruminococcaceae	0.0028	0.0010	0.0026	0.0012	Intake	0.0004	0.0486
denovo6911	Family Ruminococcaceae	0.0036	0.0016	0.0039	0.0013	Gain*Intake	0.0007	0.0492
denovo69598	Family Ruminococcaceae	0.0029	0.0014	0.0010	0.0033	Gain*Intake	0.0006	0.0495
denovo25283	Family Ruminococcaceae	0.0411	0.0304	0.0258	0.0458	Intake	0.0057	0.0497
denovo359	Family Ruminococcaceae	0.0196	0.0112	0.0234	0.0073	Intake	0.0047	0.0499
denovo8377	Order Bacteroidales	0.0024	0.0012	0.0027	0.0009	Gain*Intake	0.0006	0.0494
denovo40361	Order Clostridiales	0.0031	0.0018	0.0034	0.0015	Gain*Intake	0.0007	0.0491
denovo43715	Order Clostridiales	0.0118	0.0095	0.0148	0.0066	Intake	0.0023	0.0492
denovo51765	*Oscillospira*	0.0035	0.0088	0.0033	0.0090	Gain	0.0016	0.0484
denovo26903	*Prevotella*	0.0034	0.0103	0.0114	0.0022	Gain	0.0022	0.0494
denovo23534	*Prevotella copri*	0.0121	0.0042	0.0131	0.0031	Gain*Intake	0.0036	0.0491

^a^Data is shown as LSMeans.

^b^ *P* values indicate groups that differ (*P* < 0.05).

## Discussion

The function of the GIT is essential for the overall health and well-being of ruminants. In addition to many other host-microbiome interactions (Williams and Coleman 1997; McDonald et al. 2002; Chung et al. 2012), the various cellulolytic, metabolic, and fermentative functions supplied by the microbial communities throughout the bovine GIT contribute towards the overall energy and nutritional input to the host, and therefore have a significant effect on host maintenance, growth, and performance. Many microbial studies on bovine feed efficiency have focused on ruminal associations; however, the multiple sections within the GIT are specific in function and microbial community population and diversity (de Oliveira et al. 2013; Frey et al. 2010). The long large intestine and colon in cattle is the site of post-ruminal degradation of cellulose and starch, and is thought to be significant in animal digestion, particularly as it pertains to diet (Armstrong and Smithard 1979). Thus, determining host-microbe associations within the entire GIT from the sampling of digesta from one site will not accurately represent the nutritional and energy status of the host. The examination of the microbial associations with feed efficiency outside of the rumen, such as the colon, will aid in the comprehensive understanding of feed efficiency in beef cattle.

Compared to other sections within the GIT, the microbial abundance and diversity of the colon contents is far greater, even compared to that of the rumen (Myer et al. 2015; de Oliveira et al. 2013; Reti et al. 2013). To accurately capture most of the bacterial OTUs within the rectal contents of the steer, the study normalized the samples to a depth of 100,000 sequences/sample. This depth was estimated from colonic content sample rarefaction curves, as well as estimations from previous GIT studies, which have acquired adequate coverage (Jami and Mizrahi 2012). The current study was able to recover approximately 97% of all OTUs calculated at 0.03% dissimilarity, as determined by Good’s coverage estimator. Based on the coverage estimates of the current study, the colonic digesta sample rarefaction curves, and the coverage estimates from previous studies, the 100,000 sequences/sample achieved was determined to be satisfactory for colonic microbial community analyses.

Next-generation sequencing (NGS) technologies have allowed for greater sequencing depth of environmental niches and greater identification of OTUs, specifically when compared to previous studies examining the beef cattle GIT (de Oliveira et al. 2013; Reti et al. 2013). The caveat to this approach is that these technologies are limited to shorter read lengths than traditionally produced by cloning and sequencing of full-length 16S rRNA genes. The 16S rRNA gene includes multiple regions containing variable sequence interspersed with conserved regions, and determination of target variable regions is dependent upon the niche examined. In this study, the V1–V3 variable regions were selected to interrogate the colon content bacterial communities. However, the alpha-diversity analyses across the feed efficiency groups revealed no differences in the number of OTUs, richness (Chao1), diversity (Shannon Index) or evenness (equitability), despite the depth and increased detection of OTUs. The weighted and unweighted UniFrac PCoA also reflected the similarities of the bacterial communities within the colon between feed efficiency groups, as the microbial populations within the colon did not cluster by host phenotype. The weighted and unweighted UniFrac results are dependent upon and a result of the phylogenetic divergence between the OTUs. The lack of observable differences between communities at the phylogenetic level may be anticipated, where differences are usually observable in the feces of cattle fed different diets (Kim et al. 2014), but may not be detectable when examining finer nutritional changes within cattle on the same diet. This may further indicate that variation within the colon microbial comminutes as a function of differing feed efficiency arises from changes in OTU and relative taxonomic abundances, rather than the phylogenetic diversity of the community. These specific changes may have profound effects on the host. However, the observed similarities between the microbial comminutes in the colon may also partly be a result of host-specificity, which has been demonstrated in the rumen (Weimer et al. 2010).

The 16S sequences within the colon samples largely belonged to the phyla Firmicutes, Bacteroidetes, Spirochaetes, Tenericutes, Proteobacteria, Actinobacteria, and Fibrobacteres, which are present in the majority of gut-associated phylotypes in a variety of mammals (Ley et al. 2008; Shanks et al. 2011). The ubiquitous nature of these phyla within mammals suggests their critical role in the microbial ecology of the mammalian gut. There were observable differences at the phylum level, especially within the ADG_High_–ADFI_Low_ group. Shifts between the Firmicutes:Bacteroidetes ratio were evident, with an increase in the ratio within the ADG_High_–ADFI_Low_ group. Although the variable proportions of the phyla Firmicutes and Bacteroidetes were not significant, shifts in the ratio have been associated with obesity in humans (Turnbaugh et al. 2009; Ismail et al. 2010), as well as in the cattle rumen regarding energy harvesting and correlated increases of fat (Jami et al. 2014). However, any putative role of variation in these phyla in the lower GIT of beef cattle is unclear. In addition, the abundance of Firmicutes was comparable to that of the cecum and feces in similar studies, where members of the families Ruminococcaceae, Lachnospiraceae and Clostridiaceae dominated (de Oliveira et al. 2013). It is likely that these taxa also contribute to further downstream feed fermentation in the large intestine.

At the sub-phylum level, the colon content samples were dominated by orders Clostridiales and Bacteroidales, families Ruminococcaceae, Lachnospiraceae, Bacteroidaceae, and Clostridiaceae, as well as genera *Prevotella*, *Ruminococcus*, and *Coprococcus*. These taxa are commonly found within microbial communities across the GIT, and are also identified in the large intestine of steers (de Oliveira et al. 2013). These profiles are also similar to those identified in the fecal content of cattle (Kim et al. 2014; Jeong et al. 2011).

The colon content from the four feed efficiency groups revealed significant differences in the relative abundance of specific taxa. The putative functions of the identified organisms may provide some insight as to their potential association with regard to feed efficiency in beef cattle. *Prevotella* has commonly been found in cattle feces and has been associated with differences in diet (Durso et al. 2012). Its abundance was positively correlated with corn-based diets, and was nearly absent in cattle fed silage/forage (70% corn silage and 30% alfalfa haylage) (Kim et al. 2014), and may play an important role in the fecal microbial comminutes of feedlot cattle fed concentrate diets. Although found in the microbial populations of cattle feces, not much is known as to the function of the family Mogibacteriaceae in the gut. However, these populations in humans and mice have clustered with other organisms that are associated with lower body mass index (BMI) (Goodrich et al. 2014), as well as changes in the diet of non-obese diabetic (NOD) mice supplemented with cellulose, pectin, and xylan (Toivonen et al. 2014). Further study of Mogibacteriaceae is warranted in order to better determine its association with feed efficiency in cattle. The presence of *Faecalibacterium* in preweaned dairy calves has been associated with weight gain and the incidence of diarrhea, with greater abundance being linked with higher weight gain and less diarrhea (Oikonomou et al. 2013). The observed greater abundance in the large intestine of preweaned dairy calves may be important for maintaining proper BW and reducing enteric infections early in life. This butyrate-producing taxa is also greater in the feces of obese children than for non-obese children (Balamurugana et al. 2010), suggesting its potential function in terms of cattle health and performance. *Succinivibrio* species play an ecologically important role as starch digesters and ferments glucose with production of acetic and succinic acids (Bryant and Small 1956). Appropriately, these species are usually found in the rumen of cattle fed diets containing high levels of rapidly fermented carbohydrates. Finally, the butyrate-producing *Pseudobutyrivibrio* are core genera found to be ubiquitous in the steer GIT. Not only is its butyrate production implicated as a primary metabolic fuel for enterocytes (Scheppach et al. 1995), but the *Butyrivibrio* and *Pseudobutyrivibrio* species have gained greater interest because of their strong xylan-degrading abilities (Morgavi et al. 2013).

The relative abundance of significant OTUs within the digesta of the terminal colon content revealed many associations previously mentioned within the taxonomic assessment, however, several additional identifications were of interest. The family Ruminococcaceae contains organisms that are known to be cellulolytic, as well as active in acetate, formate, and hydrogen production (Biddle et al. 2013). A contribution by fiber-digesting bacteria is anticipated from residual feed from the rumen and thus may also contribute to downstream feed fermentation in the large intestine. Genera within the family Rikenellaceae produce acetate, succinate, and propionate as fermentation products and are found in high abundance within the large intestine and feces of cattle (Jeong et al. 2011; Kong et al. 2010; Abe et al. 2012). The family Rikenellaceae has previously been associated with forage diets (Petri et al. 2013). This finding, along with the observation that Rikenellaceae genera clustered with other genera including *Fibrobacter* in diets containing fractions of bermudagrass hay rather than wheat fractions, hint that the genera within Rikenellaceae may be involved in structural carbohydrate degradation (Pitta et al. 2010). The association of *Coprococcus* with high grain diets was also implicated in this study (Kim et al. 2014), and its butyrate production may additionally contribute towards the enterocyte energy pool, similar to that of *Butyrivibrio*, *Pseudobutyrivibrio*, and *Faecalibacterium*. *Oscillospira* are also found in high grain diets, and cattle fed high starch diets can have high bypass starch from the rumen (Wells et al. 2009). The abundances and variability of *Oscillospira* in the colon between feed efficiency groups may be associated with differing levels of bypass starch (Kim et al. 2014). Lastly, *Blautia* spp. are common inhabitants of the GIT and feces of cattle and goats, and are ubiquitous among humans and other mammals, although at low abundance (Eren et al. 2015). Within the family Lachnospiraceae, the genus *Blautia* can provide energy to their host from polysaccharides that other gut microorganisms cannot degrade (Biddle et al. 2013), and thus may be integral towards the metabolic capacity of the host.

The taxa and OTUs that were identified in the study were also analyzed to determine whether their association with feed efficiency differed individually by gain (ADG) or intake (ADFI), or their interaction. At both levels of analysis, the significantly identified taxa and OTUs were associated with either ADFI or the interaction of ADG and ADFI. The gradual association with ADFI over ADG as digesta moves distally through the GIT may be expected as the metabolic function and capacity of the tissues change, from the rumen to that of the colon (Myer et al. 2015). In addition, digestible fiber that is not degraded in the rumen or escapes digestion in the rumen, partially due to high ADFI, becomes available for further processing in the lower GIT (Tan et al. 2002). Only two taxa/OTUs associated primarily with ADG alone. These included the genera *Oscilliospira* and *Prevotella*. Their association with ADG may be explained by their abundance in the rumen and potential association with bypass starch from the rumen (Wells et al. 2009). However, it is important to note that the association with ADG may also be a result of residual DNA from other sections of the GIT.

## Conclusions

The taxa and OTUs identified in this study as associating with differences in feed efficiency have the potential to affect beef cattle feed efficiency based on their putative functions relating to cellulolytic and metabolic activities in the colon. No phylogenetic differences were observed between communities of differing feed efficiency phenotypes, although, significant differences were identified when examining the relative abundance of specific taxa and OTUs. This study suggests differences in the colon microbial communities at the 16S level in cattle that vary in feed efficiency. However, it is still not clear whether changes in the microbial community are contributing to differences in feed efficiency of the host or if host factors are driving changes in the microbial community.

The associations between feed efficiency and microbial populations in the bovine rumen have been reported (Myer et al. 2015), but knowledge of their role in the distal GIT is lacking. Aided by the coverage and depth of this study, specific and significant taxa and OTUs were identified and correlated with differing feed efficiency phenotypes, ADG, and ADFI. Additional study, including functional analyses based on the putative functions of the significant taxa and OTUs identified in this study, as well as a metagenomic approach may complement existing data and allow for greater resolution analyses, respectively. Many factors influence the feed efficiency of the host, and although important for the energy production and nutrient supply to the animal, the study of feed efficiency must occur in addition to and beyond the rumen in order to fully understand the processes contributing to efficient beef cattle.

## Methods

### Experimental design and colon sampling

Similar to Myer et al. (2015), steers selected for this study came from a population of cattle being developed to have a high percentage of the following breeds: Angus, Beefmaster, Brahman, Brangus, Braunvieh, Charolais, Chiangus, Gelbvieh, Hereford, Limousin, Maine Anjou, Red Angus, Salers, Santa Gertrudis, Shorthorn, Simmental, South Devon, and Tarentaise. Each year heifers and cows were artificially inseminated with semen from prominent industry bulls of their dominant breed. This resulted in offspring ranging from 50 to 75% of the same breed as their sire with the exception of Angus and Hereford, which ranged from 50 to 100% of the same breed as their sire. Individual feed intake was measured using an Insentec feeding system (Marknesse, The Netherlands). Steers were fed a ration (dry matter basis) of 57.35% dry-rolled corn, 30% wet distillers grain with solubles, 8% alfalfa hay, 4.25% supplement (containing 0.772 g/kg monensin), and 0.4% urea. Individual feed intake (FI) and body weight (BW) gain were measured over a 63-day period (Myer et al. 2015; Lindholm-Perry et al. 2013). Steers were selected from two contemporary groups. Group 1 (*n* = 148) were spring-born calves that were 371 ± 1 d of age and weighed 522 ± 4 kg at the start of the feed intake measurement. Group 2 (*n* = 197) were fall-born calves that were 343 ± 1 days of age and weighed 448 ± 4 kg at the start of the feed intake measurement. At the end of each feeding period, steers were ranked based on their standardized distance from the bivariate mean (average daily gain [ADG] and average daily feed intake [ADFI]) assuming a bivariate normal distribution with a calculated correlation between ADG and ADFI. Four steers with the greatest deviation within each Cartesian quadrant were sampled (*n* = 16/group). In the event a sire breed was over represented within a quadrant a steer with the next highest rank of a different breed was selected. The result was a 2 × 2 factorial design consisting of high and low ADFI, and high and low ADG (Myer et al. 2015). Steers were allowed ad libitum access to feed within 1 h prior to harvest. At the end of the feeding period, steers were harvested, and approximately 15 mL of colonic digesta at the rectum was sampled. The 2 feeding studies yielded 32 animals for analysis. Digesta was collected from the terminal colon adjacent to the rectum at harvest. Samples were individually stored in buffered peptone water (BPW, pH 7.0) +15% glycerol stock for processing and kept at −70°C for long-term storage post-processing.

### DNA extraction, amplification and sequencing

DNA was extracted from colon samples using a repeated bead beating plus column (RBB + C) method (Yu and Morrison 2004). Briefly, 0.3 g of sample was centrifuged for 5 min at 16,000×*g* to pellet solids including bacterial cells, and then resuspended in 0.2 mL TE (Tris–EDTA, pH 8.0) buffer. Cell lysis was achieved by bead beating 0.15 g of the resuspended sample in ZR BashingBead Lysis Tubes (Zymo Research Corp, Santa Ana, CA, USA) using the TissueLyser II system (Qiagen, Hilden, Germany) for 3 min at 21 Hz, in the presence of 4% (w/v) sodium dodecyl sulfate (SDS), 500 mM NaCl, and 50 mM EDTA. After mechanical and chemical cell lysis, 10 M ammonium acetate (260 µL) was used to precipitate and remove the impurities and SDS, followed by equal volume isopropanol precipitation for the recovery of the nucleic acids. Supernatants were treated with 2 µL RNase (10 mg/mL) and proteinase K (QIAamp DNA Stool Mini Kit), followed by the use of QIAamp columns from the Qiagen DNA Stool Mini Kit (Qiagen, Hilden, Germany). Genomic DNA concentration was determined using a Nanodrop 1000 spectrophotometer (ThermoScientific, Wilmington, DE, USA).

Amplicon library preparation was performed by PCR amplification of the V1–V3 region of 16S rRNA gene, using modified universal primers 27F (5′-Adapter/Index/AGAGTTTGATCCTGGCTCAG) and 519R (5′ Adapter/Index/GTATTACCGCGGCTGCTG) including TruSeq^®^ adapter sequences and indices, as well as AccuPrime™ Taq high fidelity DNA Polymerase (Life Technologies, Carlsbad, CA). Amplification consisted of 23 cycles, with an annealing temperature of 58°C. Products were purified using AmPure^®^ bead purification (Agencourt, Beverly, MA, USA) and all libraries were quantified by the PicoGreen^®^ dsDNA quantitation kit (Invitrogen, Carlsbad, CA, USA) and by real-time PCR on the LightCycler 480 system (Roche, Mannheim, Germany). The PCR amplicon libraries were sequenced using the 2 × 300, v3 600-cycle kit and the Illumina MiSeq^®^ sequencing platform (Illumina, San Diego, CA, USA).

### Sequence read processing and analysis

All sequences were processed using the QIIME-1.8.0 software package. Paired reads were joined using fastq-join (Aronesty 2011) and filtered for quality (≥Q25) using the Galaxy server (Blankenberg et al. 2010). Sequences that contained read lengths shorter than 400 bp were removed and adapters/index sequences were trimmed. Chimeric sequences were checked using ChimeraSlayer (Haas et al. 2011). All cleaned sequences were classified into taxa using the Greengenes 16S rRNA Gene Database (DeSantis et al. 2006). Operational taxonomic units (OTUs) were calculated using the uclust program (0.03 dissimilarity; Edgar 2010). After calculating richness for each quadrant, singletons were removed from further diversity analyses. Based on rarefaction curves, the number of OTUs was normalized via subsampling 100,000 sequences from each colon sample. A phylogenic tree was built with FastTree (Price et al. 2010) to determine alpha- and beta-diversity metrics.

### Statistical analysis

All analyses were conducted using SAS 9.4 (SAS Inst. Inc., Cary, NC, USA). The mean abundances (*n* = 8) of data metrics and each taxon were compared among the feed efficiency groups using a model of contemporary group and Cartesian quadrant [high ADG, high ADFI (ADG_High_–ADFI_High_); high ADG, low ADFI (ADG_High_–ADFI_Low_); low ADG, low ADFI (ADG_Low_–ADFI_Low_); low ADG, high ADFI (ADG_Low_–ADFI_High_)] as fixed effects. Significant differences were determined at *P* < 0.05 with the Benjamini–Hochberg method used for multiple-testing corrections (Benjamini and Hochberg 1995). Multiple-testing corrections were made for the number of phyla, the number of OTU groups, and other classified taxa groups. Linear contrasts were then applied to significant quadrants to separate whether microbial populations varied by low vs. high ADG, low vs. high ADFI, or their interaction (*P* < 0.05). Principal coordinates analysis (PCoA) was performed using weighted and unweighted UniFrac analyses (Lozupone and Knight 2005).

## Abbreviations

ADFI: average daily feed intake
ADG: average daily gain
BW: body weight
FI: feed intake
GIT: gastrointestinal tract
OTU: operational taxonomic unit
